# The Potential Biotherapeutic Targets of Contrast-Induced Acute Kidney Injury

**DOI:** 10.3390/ijms24098254

**Published:** 2023-05-04

**Authors:** Alice Shasha Cheng, Xiaogang Li

**Affiliations:** 1 Department of Internal Medicine, Mayo Clinic, Rochester, MN 55905, USA; cheng.shasha@mayo.edu; 2 Department of Biochemistry and Molecular Biology, Mayo Clinic, Rochester, MN 55905, USA

**Keywords:** contrast-induced acute kidney injury (CI−AKI), contrast media, inflammation, oxidative stress, biotherapeutic targets

## Abstract

Contrast-induced acute kidney injury (CI−AKI) is manifested by an abrupt decline in kidney function as a consequence of intravascular exposure to contrast media. With the increased applicability of medical imaging and interventional procedures that utilize contrast media for clinical diagnosis, CI−AKI is becoming the leading cause of renal dysfunction. The pathophysiological mechanism associated with CI−AKI involves renal medullary hypoxia, the direct toxicity of contrast agents, oxidative stress, apoptosis, inflammation, and epigenetic regulation. To date, there is no effective therapy for CI−AKI, except for the development of strategies that could reduce the toxicity profiles of contrast media. While most of these strategies have failed, evidence has shown that the proper use of personalized hydration, contrast medium, and high-dose statins may reduce the occurrence of CI−AKI. However, adequate risk predication and attempts to develop preventive strategies can be considered as the key determinants that can help eliminate CI−AKI. Additionally, a deeper understanding of the pathophysiological mechanism of CI−AKI is crucial to uncover molecular targets for the prevention of CI−AKI. This review has taken a step further to solidify the current known molecular mechanisms of CI−AKI and elaborate the biomarkers that are used to detect early-stage CI−AKI. On this foundation, this review will analyze the molecular targets relating to apoptosis, inflammation, oxidative stress, and epigenetics, and, thus, provide a strong rationale for therapeutic intervention in the prevention of CI−AKI.

## 1. Introduction

The maintenance of kidney function is essential for the excretion of waste products and proper body metabolism. Renal tubules are the primary functional component of the kidneys, and damage to them can lead to renal impairment. Despite the likelihood of the occurrence of injury to renal tubules as a result of exposure to toxins, hypoxia, and some drugs [1], their sensitivity to these damage-inducing agents depends on the cell type, position in the nephrons, the nature of injury, and local vascularization [2]. Dysfunctional renal cells are associated with cell death, proliferation, and inflammation; however, they also possess the ability to recover during acute kidney injury syndrome.

Acute kidney injury (AKI) is defined as a sudden impairment in kidney function, i.e., in a period of days or one week. The etiology of AKI is multifactorial and includes major surgery, pharmacological treatments, radiocontrast exposure, and decreased renal perfusion pressure [3,4]. Regarding incidence, AKI can develop into a critical complication in patients admitted to hospital and is observed in more than 50% patients in intensive care units (ICUs) [3]. In ICUs, AKI is common among patients and is associated with significant mortality and morbidity. Therefore, early diagnosis is essential for recovery. In a clinical context, factors such as a reduction in urinary output (oliguria) and an increase in serum creatinine levels (a marker of kidney excretory function) are what determine kidney function [4]. However, clinical diagnoses are usually delayed or missed in AKI patients because of the absence of the appearance of renal function deterioration [5]. As a consequence, the underlying symptoms often worsen, eventually sometimes even causing death. Moreover, studies have shown that even routine measurements of urea, nitrogen, and creatinine in the blood serum, as well as urine production, cannot be used to recognize the early stages of structural damage and renal dysfunction [6]. An increase in serum creatinine is related to filtration, but this measurement cannot be used to detect tubular disturbances in AKI because, in general, there is no appearance of any such symptom in AKI. Some non-renal etiologies, including nutrition intake and muscle mass, may cause changes in creatinine levels, although this is not specific to AKI. Overall, it is not surprising that there are no proper strategies to diagnose or limit AKI due to a lack of overt symptoms. No doubt an innovative diagnostic strategy shall be developed and biomarkers identified for early AKI diagnosis, which will facilitate the introduction of pharmaceutical targets and early intervention in the treatment of AKI.

The pathophysiology of AKI is complicated, and its persistent progression can result in irreversible renal damage or ischemia. First, alterations in renal prefusion and renal autoregulation during the progression of AKI engender renal vasoconstriction [7]. Second, intra-tubular blockage due to cell desquamation results in the dysregulation of excretion metabolism that ultimately affects the electrolyte balance in tubular glomerulus [8]. Third, cell death, including necrosis and apoptosis, exacerbates tubular dysfunction in AKI [9]. Last, the occurrence of local inflammatory mediators could also cause vascular congestion and interstitial inflammation [10]. At the individual cell level, a decrease in cell polarity, cytoskeletal integrity loss, displacement of adhesion molecules and membrane proteins, impairment in the brush border of proximal tubules, and necrosis of partial tubular epithelial cells largely contribute to AKI pathologies [11]. In more detail, the desquamation of non-viable and viable cells breaks the barrier between peritubular interstitium and filtrate, thus increasing its permeability and causing back-leak into the interstitium. Furthermore, the breakdown of the renal tubular epithelial barrier due to widespread TEC necrosis also exaggerates the host’s immune response [7]; it increases the secretion of vasoactive and inflammatory mediators, which could contribute to vasoconstriction and persistent inflammation. Damage associated molecular patterns (DAMPs) and associated factors further promote inflammatory responses post tissue injury. As epithelial barrier dysfunction is the main source of DAMPs and associated factors, it is considered to actively contribute to vigorous renal tubular inflammation and injury through DAMPs [12]. This ultimately culminates in the intratubular obstruction that further promotes the interaction between cellular debris and proteins, such as fibronectin in the lumen, thus extending the damage phase in AKI [7].

While diagnosis of AKI is difficult, imaging-based examinations remain the most suitable and only option. During imaging examinations, such as magnetic resonance imaging (MRI) and computed tomography (CT), patients are treated with a contrast media (CM) to highlight features in the image that can aid in achieving an accurate diagnosis [13]. However, it has been ascertained that contrast media or some drugs are freely filtered by renal glomeruli and excreted by the kidneys. Based on previous findings, all formulations of CM have been shown to be cytotoxic in vitro [14]. Again, it has been well established that radiocontrast agents have an association with AKI [15]. Importantly, the use of CM is more closely related to contrast-induced acute kidney injury (CI−AKI), which is defined as an exacerbation of renal dysfunction or a serious onset of new complications after the administration of CM [16]. The administration of CM can be intravenous, oral, or intra-arterial, based on the pathological conditions [17]. CI−AKI is not entirely dependent on the dosage of iodinated contrast media. Though for most examinations, 300 mg/mL of iodine is sufficient, the dosage may increase for clinical tests, such as CT angiography. However, the risk of CI−AKI differs from patient to patient and increases with the frequency of usage, aging, and in patients with diabetes, independent of dose concentration [18]. So, it is impossible to determine a particular dose of iodinated contrast media that would become toxic to patients. In general, 300 mg iodine/mL can be considered an appropriate dose for administration [19]. CI−AKI is the most severe form of AKI and belongs to one of the most devastating clinical complications, i.e., acute renal failure. It has been reported as the third most common cause of renal insufficiency among patients admitted to hospital, with a high risk of mortality. In this review, we will discuss the potential molecular mechanisms and the biological targets of CI−AKI, as well as the potential biomarkers of CI−AKI.

## 2. Molecular Mechanisms of CI−AKI

**Figure 1:**
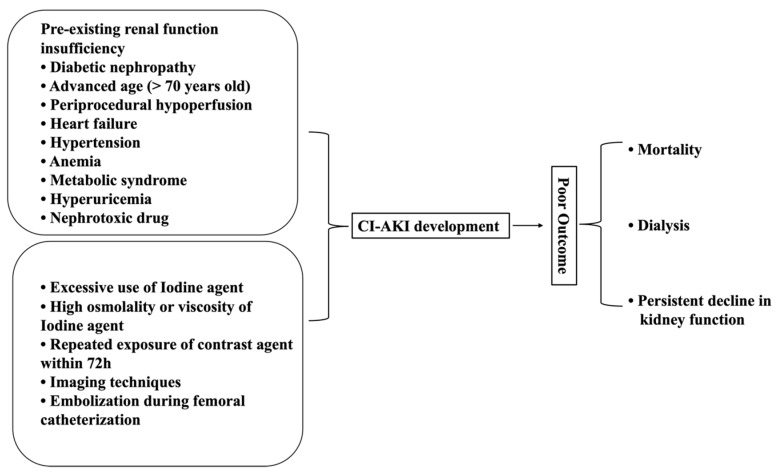
Risk factors for CI−AKI. The pre-existing renal insufficiency and inappropriate use of contrast agents in imaging techniques favors the incidence of CI−AKI. The lack of uniform diagnostic criteria and early intervention further accelerates the development of CI−AKI and decline in kidney function.

Because of the frequent use of contrast agents, CI−AKI is becoming a common disease among the various kinds of kidney injury [16,20]. Reports have demonstrated that chronic kidney disease (CKD), diabetes mellitus, and aging are the well-known risk factors that readily use contrast media and exhibit a significant effect on the incidence of CI−AKI (Figure 1). CI−AKI is identified by a significant raise in serum creatinine up to 44 μmol/L, 0.5 mg/dL, or of 25% from baseline within 48–72 h after injection of contrast media, which excludes the possible alternative etiology of CI−AKI [21]. The misuse or inappropriate use of contrast media predisposes the pathological mechanism in CM induced renal toxicity that mainly includes apoptosis, inflammation, and oxidative stress [22,23,24].

**Figure 2:**
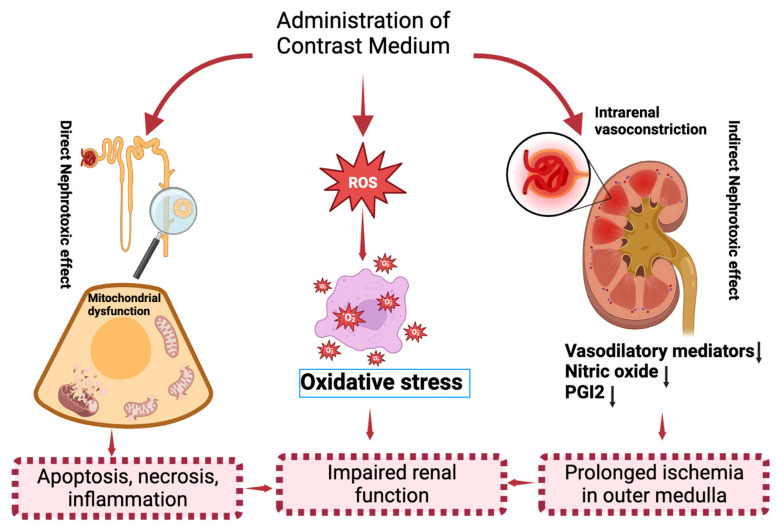
Pathophysiology of CI−AKI. Pathogenesis of CI−AKI consists of 3 mechanisms: direct effect, indirect effect, and the generation of ROS. The direct effects are mediated by a direct cytotoxicity of CM to nephron, leading to mitochondrial dysfunction, cellular apoptosis or necrosis and interstitial inflammation, resulting in tubular injury. Indirect effects are that CM could alter renal hemodynamics, leading to intrarenal vasoconstriction, contributing to medullary hypoxia. The indirect effects are mediated by an increase in vasoconstrictive mediators, including renin, angiotensin II, and endothelin, along with a decrease in vasodilatory mediators, including nitric oxide (NO) and PGI2, leading to altered renal hemodynamics and resulting in intrarenal vasoconstriction and medullary hypoxia. CM can also generate ROS, resulting in oxidative stress, and a decrease in anti-oxidant enzyme activity with various complex mechanisms to impair renal function. CM, contrast media. PGI2, prostaglandin I2. ROS, reactive oxygen species.

The mechanisms of CI−AKI have not been completely elucidated. The current proposed mechanism includes both direct and indirect effects that generates reactive oxygen species (ROS) (Figure 2). In direct effects, CM with high osmolality can directly cause cytotoxicity in nephrons, including renal tubular epithelial cells and endothelial cells, leading to mitochondrial dysfunction, cellular apoptosis or necrosis, and interstitial inflammation [12]. In indirect effects, CM can severely derange hemodynamics that causes intrarenal vasoconstriction leading to medullary hypoxia [12]. Medullary hypoxia has been studied to enhance ROS formation in renal cells, resulting in oxidative stress and mitochondrial dysfunction [13]. Hypoxic medullary prevents oxygen delivery and then lead to injury in models of renal ischemia [25]. Water-soluble CM not only shows cytotoxic effects but also causes renal damage because of different osmolarity to the surrounding tissue [26]. Additionally, higher osmolarity of CM solutions is closely related to high cytotoxicity, especially when the CM exposes to hyperosmolar environments, such as tubules and medulla, where water is absorbed by tubules and enter the medulla, resulting in an increase in tubular fluid viscosity [27]. The decrease in the urine flow rate is accompanied with tubular pressure increase, then promoting the medullary hypoperfusion [26]. In addition, CM can also promote excessive ROS production or reduce antioxidant enzyme activity, resulting in an increased oxidative stress environment that impairs renal function [13]. Thus, oxidative stress and mitochondrial dysfunction play important roles in the pathophysiology of CI−AKI [13], which are potential targets for the development of strategies in the prevention of CI−AKI.

Apart from the evidence that demonstrates oxidative stress and mitochondrial dysfunction to be associated with CI−AKI, a high degree of cellular apoptosis and damage can be observed in endothelial cells exposed to contrast media. A previous study has shown that contrast can release free iodine during the imaging procedure and cause necrosis of partial tubular epithelial cells, thus prompting an injury to surrounding endothelium [16,27]. Damage in the endothelial cells and nephron tubules can then lead to the formation of free radicals, promote apoptosis and, inflammation in CI−AKI [28].

Apoptosis, in general, can be regulated by intrinsic signaling and extrinsic receptor pathway [29]. Apoptotic extrinsic signaling pathway is activated when extracellular ligands, including Fas−L (Fas ligand), TNF (tumor necrosis factor), TRAIL (TNF-related apoptosis-inducing ligand), binds with extracellular domain of the DR (transmembrane receptors) and TRAIL receptors of the cell surface [30]. Contrastingly, the intrinsic mechanism of apoptosis is mainly associated with mitochondria [31]. Various kinds of extra and intra-cellular stresses, such as irradiation, contrast medium, cytotoxic drugs, and oxidative stress (ROS production), are the main triggers for the intrinsic pathway [32,33]. Proteins that are associated with intrinsic pathway includes Bcl−2 (B-cell lymphoma protein 2, Nox (Phorbol-12-myristate-13-acetate-induced protein 1), Bcl−w (Bcl−2-like protein), and Caspase−9 (Cysteinyl aspartic acid-protease-9), Myc (Oncogene Myc), etc. [33]. Mechanistically, it is the balance between the pro-survival (Bcl−2) and pro-apoptotic members that regulates intrinsic apoptotic pathway. Distinctly, the oligomerization of Bcl−2 pro-apoptotic members (BAX, BAD, BAK) induces the permeabilization of mitochondrial outer membrane due to the loss of mitochondrial membrane potential [34]. As a consequence, cytochrome c is released onto the cytosol and executes activation of caspase 9 in the mitochondrial pathway of apoptosis. With reference to CI−AKI, it has been demonstrated that CM activates Bcl−2 pro-apoptotic members and caspase 3, thus hinting the involvement of intrinsic pathway that induces renal cell apoptosis [35]. Indeed, the intrinsic pathway is enhanced by the ROS-mediated activation of stress kinases JNK1/2 and p38 associated with mitochondrial dysfunction in CM−AKI [36]. The abolishment of apoptosis by JNK1/2 and p38 inhibitors or activation of apoptosis through supplementation of N-acetylcysteine (NAC; induces apoptosis via intrinsic pathway) further ascertains the crucial role of mitochondrial intrinsic apoptotic pathway in CI−AKI [37]. In summary, CM stimulation mediated the apoptosis signals are mainly regulated by the mitochondrial pathway or the intrinsic pathway.

The external factors, such as CM, or intrinsic factors that causes cellular damage provokes the inflammation response that then leads to the alteration of homeostasis in renal systems and within the circulation [38]. In the progress of CI−AKI, CM toxicity-induced immune response can significantly increase the migration of immune cells and accumulation of cytokines, such as TNF−α and ILs [39]. Different cellular types induces inflammation response that is the key feature in the early development of CI−AKI [40]. Various cytokines were upregulated in diabetics and are detectable in plasma [41]. The upregulation of cytokines, including TNF−α (tumor necrosis factor-alpha), IL−1 (interleukin−1), IL−18, TGF−β (tumor growth factor-beta), IFN−γ (interferon-gamma), IL−6, and IL−33, positively correlates with the progress of DM [40] and urine proteins [41]. The facilitating effects of these cytokines on CI−AKI can be direct or indirect. Dysfunction of endothelial cells driven by cytotoxic CM also activates related signaling pathways and leads to the generation of systemic inflammation, which increases the sensitivity of the kidney to local inflammation processes [40,42].

It has earlier been known that ROS and free radicals consume nitric oxide (NO) to reduce the protective effect of NO on vasodilation [14]. Contrast can also activate the defense mechanism of antioxidant factors in AKI. It has been reported that the vasoconstrictor release by iodinated contrast media exhibits cytotoxicity and oxidative stress effect on vascular endothelial cells and renal tubular [43]. The vasoconstriction usually sustains for a few hours, which will cause medullary hypoperfusion, decrease the glomerular filtration rate (GFR), and increase the viscosity of blood through the nephron [44]. It is of note that oxidative stress increases peripheral vascular resistance to affect the cardiovascular morbidity. ROS generation influences renal blood flow by regulating vasodilators and facilitating the production of vasoconstrictors [45]. However, the role of oxidative stress in the development of CI−AKI is still not confirmed.

## 3. The Potential Biomarkers of CI−AKI

Several biomarkers have been found in AKI, such as IL-18, kidney injury molecule-1, sodium/hydrogen exchange, netrophil gelatinase associated lipocalin, Neutrophil gelatinase-associated lipocalin 3, and N-acetyl-β-D-glucosaminidase [46,47]. Opposingly, various biomarkers have also been developed to detect CI−AKI at an early stage [48]. However, the ideal CI−AKI biomarkers should be precise and fast to detect the severity of dysfunction, especially for the kidney. The biomarkers should be with 100% sensitivity and specificity and are supposed to increase at the early stage of kidney injury [49]. In addition, the biomarkers of CI−AKI should be helpful for the identification of the mechanism of the disorder, as well as the severity of kidney disfunction [50].

Different kinds of markers, including growth factors, some cytokines, tubular enzymes, and adhesion molecules, have been developed for CI−AKI early diagnosis, which are also critical for the targeting using therapies at the early stage of the disease [51]. The current promising biomarkers for detecting CI−AKI at an early stage include cystatin C, a biomarker for glomerular filtration function and β2-microglobulin, α1-microglobulin, NAG, RBP, IL−18, NGAL, Netrin−1, KIM−1, clusterin, sodium hydrogen exchanger isoform, and fetuin A, which are biomarkers for tubular reabsorption function [52]. However, they are still not advocated in clinics because of lacking specificity and sensitivity. The use of single biomarkers might also not be adequate to determine CI−AKI due to kidney heterogeneity or various dysfunction conditions [49]. Further studies are needed on the use of biomarker panel for detecting, monitoring, and determining the prognosis of CI−AKI.

The potential biomarkers that may evaluate kidney damage post treatment with contrast media have been investigated in an animal model [53]. Iopromide is one component of CM, which can increase serum symmetric-asymmetric dimethylarginine (SDMA−ADMA) levels and serum creatinine (sCr) in a short time. SDMA-ADMA and sCr are associated with an increase in renal-cell apoptosis and degeneration [53]. We summarize some potential biomarkers that have been reported in Table 1.

## 4. The Biological Targets of CI−AKI

**Figure 3:**
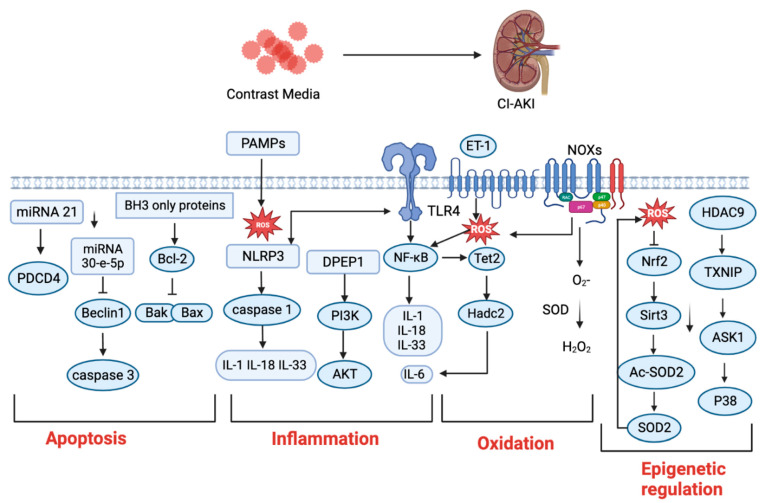
The potential biotargets associated with the CI−AKI. CM-induced apoptosis can be regulated by miRNAs (miRNA 21, miRNA 30-e-5p), and BH3, which can be through Bcl2 family (Bak and Bax), to regulate the caspase 3-mediated apoptosis in CI−AKI, suggesting that they may be potential targets. CM-induced inflammation can be regulated by Nod-like receptor pyrin containing 3 (Nlrp3) inflammasome activated caspase−1 then divide the IL−1β and IL−18 to activate inflammatory response. Brush border enzyme dipeptidase-1 (DPEP−1) is closely related to tubular reabsorption of contrast and plays an important role in CI−AKI. TLR4 has a high level of expression in contrast-induced injuries, and the activation of TLR4 can induce the NLRP3 inflammasome and NF−κB signaling pathway. CM-induced ROS can be regulated by Tet2 and NADPH oxidases (NOXs), vascular NOXs to regulate ROS generation during cellular oxidation, and by epigenetic mechanisms. For example, Tet2 signaling can specifically recruit the histone deacetylases 2 (Hdac2) to inhibit IL−6 transcription during cellular oxidation. In addition, nuclear factor erythroid 2-related factor (Nrf2) can regulate Sirt3/SOD2 in contrast-induced oxidative stress. HDAC9 promote the expression of thioredoxin-interacting protein (TXNIP), thus increasing susceptibility to CI−AKI.

The abnormal regulation of factors associated with cell apoptosis, inflammation, oxidative stress, and epigenetic regulation contributes to the majority of CI−AKI, and may be targeted for all phases of CI−AKI treatment (Figure 3). In recent years, several strategies have been developed against CI−AKI, such as the use of non-nephrotoxic genets, preventive management, and standardized treatment of iso-osmolar or low-osmolar CM [65,66,67]. However, those strategies cannot effectively reduce the rate of CI−AKI. The pharmacological prophylactic agents have also been used to reduce the incidence of CI−AKI. Increasing evidence indicates that statins have protective effect on patients at a risk of CI−AKI by inhibiting endocytosis effect of CM into tubular epithelial cells [68,69]. However, the effect of high-dose and short-term usage of statins are still needed to be confirmed as an independent preventive strategy for CI−AKI. There is a non-invasive, novel, and low-cost strategy, called remote ischemic preconditioning (RIPC), which has been proposed to apply in coronary angiography patients to effectively prevent against CI−AKI [70]. In addition, RIPC has been used in distant organs to render the kidney resistant to a subsequent sustained episode of ischemia [71]. However, still there is an immediate need to further study the potential clinical applications of RIPC in patients at risk of CI−AKI. Some antioxidant agents, such as recombinant klotho, quercetin, and febuxostat, have the potential inhibitory effect on CI−AKI, which still needs to be further evaluated for their reno-protective effects [72]. Moreover, several classes of vasodilators have been applied in CI−AKI treatment [73], while some other vasodilators, including dopamine, nifedipine, and endothelin receptor antagonists, showed no inhibitory effect on CI−AKI [74].

Although extensive research has focused on the CI−AKI, the pathophysiology involved in CI−AKI is still not clear. However, some signaling pathways and the components of those pathways may be potential biological targets for CI−AKI. Multiple adjunctive pharmacotherapies have been tested for CI−AKI prevention, but few can successfully prevent the progress of CI−AKI. A clear understanding of the molecular mechanisms in CI−AKI would be helpful to decrease the poor prognosis in CI−AKI patients at risk. Therefore, the development of novel and potential therapeutic targets is imperative for CI−AKI treatment. We will discuss the potential biological targets that are involved in the pathogenesis of CI−AKI, specifically the modulators of apoptosis, immune and inflammation, oxidative stress, and epigenetic regulation, that might introduce new CI−AKI therapeutics.

### 4.1. Apoptotic Targets

Apoptosis is an evolutionarily conserved mechanism that eliminates the unwanted cells to maintain individual’s physiological environment [75]. It is well-known that cell survival and cell death are affected by the balance between anti-apoptotic and pro-apoptotic processes [76]. Reports have demonstrated that the activity of caspases family members was significantly increased in the hypertonicity-induced cell death [77]. Evidence has also indicated that it is an important cellular event in the progress of CI−AKI because CM can induce cell injury and death [78]. The morphologic characteristics of apoptosis includes the fragmentation of DNA and other programmed cell death that are prominently observed in renal tubular, cardiac myocytes, vascular endothelial, glomerular, and smooth muscle cells of the kidneys and heart in the CI−AKI mouse models [79]. The time- and dose-dependent cell apoptosis were observed in CM-induced renal cells by activating the mitochondrial stress associated intrinsic apoptotic pathway against an extrinsic apoptotic pathway that is initiated by the binding of membrane-bound death receptors [80]. It can, thus, be speculated that development of apoptosis biomarkers could potentially detect CI−AKI before its clinical diagnosis.

At the molecular level, mitochondrial integrity is regulated by Bcl-2 family proteins [9]. The definition of Bcl-2 family proteins is mainly based on the presence of Bcl-2 homology (BH) domains and Bcl-2 family proteins can be anti-apoptotic or pro-apoptotic [81]. The organization of the BH domains determine the specific functions of individual Bcl−2 proteins. The antiapoptotic proteins, such as Bcl−XL and Bcl−2, usually have four BH domains. Proapoptotic members are classified as the multi-BH domain proteins, such as Bak and Bax, and the BH3-only proteins, such as PUMA and Bid [82], whereas the antiapoptotic protein Bcl−2 can preserve mitochondria integrity to protect cells from injury, the proapoptotic protein can permeabilize the organelles to kill cells [83]. Bak and Bax are the critical proteins in mitochondrial injury in various kinds of apoptotic model [84]. The deletion of Bax and/or Bak consistently leads to a marked resistance to tubular apoptosis in AKI [85]. The activation of Bax and Bak plays a vital role in mitochondrial injury during apoptosis which involves a striking change of mitochondrial morphology and consequent alterations of the membrane property. However, the mechanism of their activation is still not clear. Normally, Bax is cytosolic, whereas Bak resides on the mitochondrial outer membrane. During cell stress or apoptosis, Bax translocates to mitochondria, inserts to the outer membrane, and forms oligomers. Meanwhile, Bak is also activated to oligomerize. However, Bax activation may be partnered with specific proteins, such as humanin, Bid, 14-3-3 protein, p53, Bif−1, and ku70 [86]. It has recently been shown that the interaction of nucleophosmin with Bax is critical to Bax activation during metabolic stress in vitro and during ischemic AKI in vivo [87].

Caspase proteins are closely associated with cell apoptosis progress, and they usually are activated downstream of other signals [88]. During cell apoptosis, cytokeratin−18, an intermediate filament protein of simple epithelial cells, is cleaved by caspases. As a result, M30, which is the important caspase-cleaved fragment of cytokeratin−18, can be detected [89]. Importantly, cell free DNA can be detected in urine that comes from injured kidney plasma. Notwithstanding, the modulation of caspase activity can induce HSP70 activation to inhibit the rate of apoptosis and cell damage. Importantly, HSP70 has been reported as the early biomarker of AKI [90]. Elevated HSP70 levels in urine have been detected in a small study of critically ill patients before the clinical diagnosis of AKI [91]. Therefore, the caspase protein could also serve as the promising biomarkers in CI−AKI.

The small molecules microRNAs (miRNAs) contain about small, single-stranded RNA, 18–24 nucleotides long [92]. MiRNAs target mRNAs or suppress the translation progress and then regulate post-transcriptional gene expression [80]. miRNAs have differential expression patterns in renal tissues as examined by microarray assay [43]. Research has shown that miRNAs are involved in CI−AKI and some other related kidney diseases [93]. There are 11 miRNAs with a differential expression pattern in CI−AKI rat [44]. Through modifying the analysis technology, it has been further found that 22 miRNAs were downregulated, and 19 miRNAs were upregulated in the CI−AKI rat model [45]. Cellular and in vivo CI−AKI analysis showed that these aberrant expressed miRNAs are closely related to apoptosis. MiR−188 is overexpressed in both CI−AKI rat model and iodixanol-treated HK−2 cells. It targets SRSF7 and promotes the contrast-induced apoptosis [46]. MiR-30c is upregulated in CM-treated miniature pigs and inhibits kidney injury and cell apoptosis by targeting Nlip3 inflammasome [47]. Moreover, MiR-30a, -30e, and miR-188 are also upregulated in CM-induced plasma compared with normal healthy plasma [44].

However, most of the studies about kidney diseases mainly focus on the unique miRNA expression patterns [94]. In ischemia-reperfusion-induced kidney injury, the miRNA−192-5p and miRNA-30c-5p could be the potential biomarkers in urine [95]. It has been suggested that miRNA−21 prevents the cell apoptosis by regulating programmed cell death protein 4 (PDCD4) in CI−AKI [96]. In the CI−AKI rat model, CM significantly decreased the expression of miRNA21 and induced cell apoptosis in kidney tissues [96]. Thus, the expression of PDCD4 and apoptosis can be antagonized with miR-21 mimics in CI−AKI [97].

miRNA-30e-5p has been proposed as a promising new therapeutic targets against kidney diseases [98]. miR-30e-5p could be increased by urografin in HK−2 cells to induce renal cell injury via the increase in caspase 3 to promote apoptosis and concurrently, downregulating the expression of Beclin1 to decrease autophagy [93]. Therefore, an upregulation of Beclin1 or an inhibition of miR-30e-5p expression should suppress apoptosis and promote autophagy to increase cell viability in CI−AKI, which could be potential targets for CI−AKI treatment. Taken together, although the study on miRNAs in CI−AKI is still in development, current evidence suggest that miRNAs have the potential to be used as diagnostic biomarkers or novel therapeutic targets for CI−AKI.

### 4.2. Inflammatory Targets

Our immune system has the capacity to respond to pathogenic injury called inflammation; this physiological process is important to protect the organism from insults. The role of inflammation and immune response is vital in the progress of kidney injury [99]. However, the exact mechanism of inflammation in CI−AKI has not been clearly identified [99,100].

**Figure 4:**
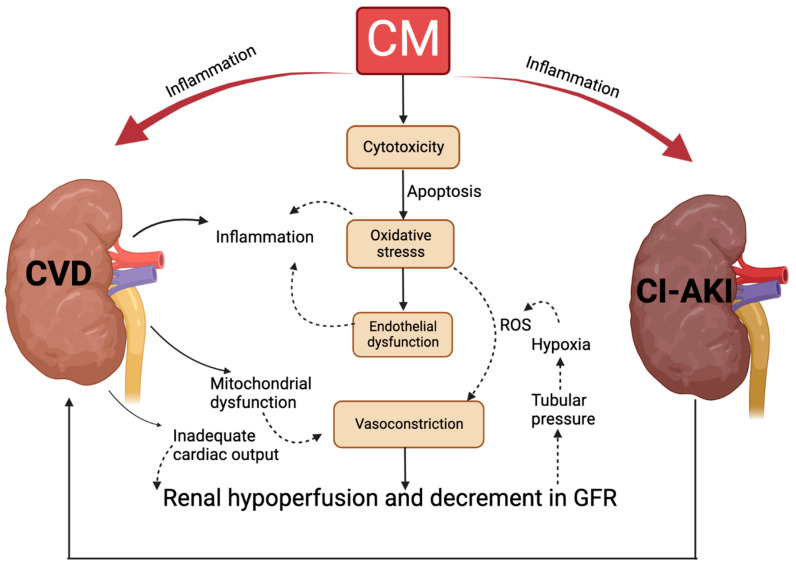
Interaction between CI−AKI and cardiovascular disorders pathophysiology. Mechanisms triggered by Contrast Media lead to CI−AKI. Their interaction promotes mitochondrial dysfunction, excessive ROS, promotes cytotoxicity and apoptosis. CI−AKI enhances CVD pathophysiology. CVD processes (indicated with black arrows) show CI−AKI/CVD potential interaction creating a feedback loop that will enhance heart and kidney malfunction. Black arrows: CVD process. Dotted arrows: CI−AKI process. ROS: reactive oxygen species.

Inflammation is also a hallmark in CI−AKI and, in that the inflammatory elements are the significant features in the patients with high risk of CI−AKI. Stress from environment can lead to oxidative stress and ROS production that are known to regulate lipid peroxidation and the processes of inflammatory response [101]. Furthermore, ROS is the major factor that cause various inflammatory diseases, including AKI, CI−AKI, CKD, type II diabetes, cardiovascular diseases (CVD), and cancer [12,13,27]. It has been shown that CVD patients with active inflammatory progress may attribute its high-risk to developing AKI after exposing the CM [45]. In recent years, ROS and inflammatory molecules have been developed as the potential drug targets and predictors in AKI and CKD [14,15], supporting that they can also be useful drug targets and predictors in CI−AKI. The interaction between CI−AKI and cardiovascular disorders pathophysiology is shown in (Figure 4).

The dendritic cells and resident macrophages compose the major component of the intrinsic immune surveillance system in the kidney and expresses various overlapping markers, such as CD11c, CD11b, CX3CR1, and F4/80 [102]. The current understanding of the intrarenal immune system in kidney diseases remains limited and is inferred primarily from cell depletion studies. Moreover, the exact molecular mechanism which activates the resident renal phagocytes that necessitates immune response has not been clearly elucidated.

Emerging evidence indicates that a Nod-like receptor pyrin containing 3 (Nlrp3) is a critical immune sensor during CI−AKI [103,104]. It is reported that canonical Nlrp3 inflammasome activates caspase−1, which drives inflammation. Then, caspase−1 divides the IL−1β and IL−18 into active and mature forms and activate inflammatory response. However, research showed that the function of Nlrp3 in the epithelial cell compartment of the kidney is primarily non-canonical and serves as the platform for regulation of apoptotic cell death and caspase−8 activation [103]. Therefore, the activation of canonical Nlrp3 inflammasome in the CI−AKI kidneys is most likely due to the circulating leukocytes recruited to the injured kidney or renal phagocytes. Again, it has been reported that canonical Nlrp3 inflammasome could be activated by the contrast in macrophages and then regulate inflammation progress in CI−AKI [105]. Unfortunately, Nlrp3−mediated tubular cell death is not involved in CI−AKI [106].

In addition to the role of Nlrp3, the brush border enzyme dipeptidase−1 (DPEP−1) is responsible for the tubular reabsorption of contrast and is another critical factor in the pathogenesis of CI−AKI. After the glomerular filtration, DPEP−1 could be the luminal receptor for various media or drugs entering the urine. Importantly, the DPEP−1 inhibitor cilastatin is a single agent in clinical trials for CI−AKI treatment [106]. On the other hand, NLRs participate in the formation of some signaling complexes and causes damage. For example, Nlrp3 inflammasome is a complex which can activate caspase−1 and then promotes IL−1beta, IL−18, and IL−33 maturation and release [107]. It has been shown that Nlrp3-dependent inflammatory response is one of the several steps accompanied in the immune surveillance of CI−AKI [108]. Contrary to the above results, IL−22 can alleviate renal injury and fibrosis in DN by inhibiting the Nlrp3/caspase−1/IL−1beta inflammatory response pathway [109]. Another study indicated that resolvins inhibited the formation of Nlrp3 inflammasome and NF−κB pathway and recruited inflammatory cells, thereby relieving oxidative stress and alleviating DN [110], which provided indirect evidence to the participation of Nlrp3 in CI−AKI. Therefore, targeting Nlrp3 inflammasome and DPEP−1 may be useful as an adjunct to the current standard of care for the prevention of CI−AKI.

A recent study showed that oxidative stress (ROS and H_2_O_2_) promoted the expression of Toll-like receptor 2 (TLR2) and Toll-like receptor 4 (TLR4) in human periphery monocytes to increase the expression of cytokines, such as IFN−gamma, IL−1 beta, IL−6, etc., in diabetes mellitus (DM) [41]. This finding provided a clue as to how DM increases pro-inflammatory response that can propagate CI−AKI incidence [111]. Some other studies indicated that in non-contrast-induced AKIs, TLRs interplay with damage-associated molecular patterns (DAMPs) or pathogen-associated molecular patterns (PAMPs), resulting in immune reactions or mediating immune responses [112]. In DM and Diabetes nephropathy (DN), DAMPs stem from dead or damaged cells, and they could be metabolites of DNA, harmful particle or ATP, such as uric acid crystals, or can be generated by radiation [41]. Heat shock protein (HSP) and high mobility group box-1 protein (HMGB−1) are the most common DAMPs in AKI [113]. DAMPs become exposed to the immune system via cytolysis, cellular excretion, or enzyme matrix releasing to promote immune response or inflammation [114]. On the one hand, innate immune cells express TLRs to recognize DAMPs, activating some signaling pathways, such as necrosis factor-kappa B (NF−κB) and MAPK, and promoting cytokine release. Some of these factors may be involved in CI−AKI.

In relation to TLRs in inflammation, TLR4 is a pattern recognition receptor which is majorly responsible for the inflammatory response [107,114,115]. Increasing evidence indicates that TLR4 plays an indispensable role in the pathogenesis of acute kidney injury [108]. TLR4 is expressed in resident renal cells, including mesangial cells, podocytes, endothelial cells, and tubular epithelial cells, which is involved in the regulation of the transcription of numerous chemokines and pro-inflammatory cytokines, leading to the renal inflammation [108]. Moreover, TLR4 is an important upstream regulator for some inflammatory pathways and functions as the critical mediator of AKI [114]. Targeting TLR4 could inhibit the progress of various kinds of AKI [107,110]. In recent years, TLR4 is discovered as a novel target in treatment of CI−AKI [107]. TLR4 is significantly increased in the contrast-induced injury model and activate the corresponding ligand in the pathological state [116]. TLR4 activation can induce the activation of downstream the Nlrp3 inflammasome and NF-κB signaling pathway, then generate the inflammatory cytokines, such as IL−1β, IL−6, and TNF−α, that exacerbates kidney injury [117]. Calcium-binding proteins showed the potential ability to activate TLR4 signaling axis [116]. Evidence showed that the calcium binding protein S100A8/A9 is the endogenous ligand for TLR4 in CI−AKI. TLR4 bind with these calcium-binding proteins and activate the TLR4/Nlrp3 inflammasome pathway to induce a serious of inflammatory cascade in CI−AKI [117,118]. Mice with TLR4 deficiency can prevent the I/R injury mediated tubular damage [118]. It is necessary to further explore the role of TLR4 receptors in CI−AKI and evaluate the possibility to avert TLR4-mediated local inflammation in the kidneys. Given the important role of TLR4 in different kinds of preclinical AKI models, the inhibition of TLR4 and its downstream effectors can serve as the novel therapeutic target for treatment of CI−AKI through preventing renal inflammation and subsequent kidney damage.

The kidney injury molecule 1 (KIM−1), also named TIM1 (T-cell immunoglobulin mucin receptor 1) or HAVCR1 (Hepatitis A virus cellular receptor 1), is a type I membrane protein which is composed by the cytoplasmic and extracellular portion [119]. KIM−1 is expressed in the liver, kidney, and spleen. KIM−1 is involved in HAV infections, autoimmunity, immune tolerance, and atopic diseases [120,121]. The mechanism of KIM−1 in immune diseases and kidney injury is different. The expression of KIM−1 is mainly found in the differentiated proximal tubular epithelial cells [119]. Because it is sensitive to ischemia, toxicity, and hypoxia, it could regenerate in the proximal tubule S3 outer medulla area after injury. Therefore, the level of KIM−1 in renal and urinary system is significantly increased during AKI [122]. Accumulating evidence has shown that KIM−1 is an early biomarker of AKI and also serves as an index that predicts the progress of renal outcome. KIM−1 is involved in the activation of ERK1/2 and STAT3 in acute renal tubular injury thus, contributing to inflammation [123,124]. Moreover, KIM−1 could bind with p85 and regulate the ULK1 phosphorylation in kidney diseases of protein overload [125]. KIM−1 is also involved in the activation of the ERK/MAPK signaling pathway to promote the repair process after AKI [126]. Taken together, this evidence suggests that KIM−1 may a potential target to subside inflammation against CI−AKI.

The hypoxia-inducible factor (HIF)−1α signaling can also be activated post CM administration in kidneys, leading to hypoxic injury [24]. Contrast-mediated ischemia shift the expression site of HIF−1α in the kidney as HIF−1α is highly expressed in proximal tubular regions and the glomerular of nephron [24,127]. High HIF−1α expression enhances autophagy to accelerate the contrast kidney injury [128]. Moreover, HIF−1α and another known factor, lncRNA nuclear enriched abundant transcript 1 (NEAT1), play important roles in the inflammation progress in CI−AKI [129]. lncRNA NEAT1 is overexpressed in the CI−AKI rat model [130]. Additionally, it has also been reported that lncRNA NEAT1 promotes cell apoptosis and cell injury by upregulating the expression of High Mobility Group Box-1 (HMGB1). Silencing of lncRNA NEAT1 strikingly suppressed the expression of HMGB1, further relieving the inflammatory response, cell injury, and apoptosis [129,131]. Therefore, inhibiting cell apoptosis, cell injury, and inflammatory response by inhibiting the HIF−1α/lncRNA NEAT2/HMGB1 signaling pathway could be a promising strategy against CI−AKI.

In DN patients and animal models, five kinds of cells, including neutrophils, lymphocytes, macrophages, dendrites, and mast cells, take part in renal pathogenic effect [58]. These cells infiltrate into the kidney and release pro-inflammatory factors, causing degradation and phagocytosis of necrotic cell fragments, and promote fibroblast proliferation and, thus, renal fibrosis. Except lymphocytes, the other four types of cells are important members of the innate immune system. Innate immune cells express pattern recognition receptors (PRRs), such as Toll-like receptors (TLRs) and NOD-like receptors (NLRs), to combine with specific ligands, such as DAMPs or PAMPs, to trigger immune responses. However, the current understanding of molecular patterns and receptors in CI−AKI is limited and can be further explored [59].

Currently, the retinoic acid (RA) signaling pathway is becoming a research hotpot because of its role in inflammatory response and immunosuppressive effect [132]. RA is the major metabolism derivative of vitamin A, that has been reported to prevent fibrosis and inflammation in renal injury. RA regulates inflammatory and oxidative stress and has been widely reported in CI−AKI [132,133,134]. Previous studies have also shown that RA regulates autophagy and promotes autophagosome maturation. RA increased the LC3-II/I ratio and decreased the level of p62 expression in kidney cells. Then, RA could protect against CI−AKI via activation of autophagy [135]. Elucidating the mechanism of the RA signaling pathway in AKI and its association with macrophages has greater clinical prospects [136]. During the AKI, reactivation of RA signaling reduce postinjury fibrosis and tubular injury, but has little effect on the damaged tubular epithelium repair in mouse kidneys. Therefore, RA signaling reactivation is a conserved response in renal injury progress and promote organisms repair and reduce injury [137]. Renal macrophages rely on mediating RA-dependent effects for kidney injury. Liposomal clodronate selectively activated macrophages skewing to its anti-inflammatory phenotype while decreasing inflammatory macrophage number [137].

### 4.3. Oxidative Stress Targets

Oxidative stress is an important cellular progress in the CI−AKI [138,139]. Increasing evidence has shown that oxidative stress plays a significant role in CI−AKI progress. Small molecules targeting factors causing oxidative stress is an important strategy against CK-AKI [24,25,26]. Among factors that lead to oxidative stress, hypoxia is a vital element for ATP generation. It can cause a decrease in oxidative phosphorylation and increase the production of free radicals in the mitochondria [76]. In contrast, endoplasmic reticulum stress plays a significant role in the pathophysiology of kidney injury and may result in plasma membrane injury and mitochondrial dysfunction [140]. Furthermore, injured mitochondria can lead to production of comparable ROS because of the catalytic iron release, that then promotes the Haber–Weiss and Fenton reaction [28]. The activity of mitochondrial enzymes can be inhibited by contrast agents in epithelial cells and then promote ATP hydrolysis to ADP and AMP. 5′-nucleotidase hydrolyzes AMP and generates the adenosine, which stimulates the production of hypoxanthine and inosine, and further produces H_2_O_2_ and xanthine by the action of xanthine oxidase [141]. Moreover, impaired mitochondria releases cytochrome c, which is closely related to apoptosis activation [122]. It has been shown that the generation of ROS plays key role in the progress of cytosolic Ca^2+^ overload by endoplasmic reticulum stress [40]. This explains how ROS production is responsible for the CI−AKI development. Due to the lack of plasma membrane integrity, adjacent cells and renal tubular epithelial cells can be directly injured by the excessive ROS. However, it is necessary to further investigate in detail how the ROS overproduction is induced by contrast.

Additionally, the intrinsic apoptosis pathway and stress kinases, such as p38 MAPK stress kinases, c-Jun N-terminal kinases, and caspases can be activated by ROS, and then can cause renal cell apoptosis [23]. Increasing evidence revealed that free radicals play a crucial role in renal vasoconstriction. Oxidative peroxynitrite is produced by the reaction between ROS, superoxide, and NO. The oxidative peroxynitrite will inhibit the NO bioavailability and then lead to significant damage to renal cells [29]. In addition, recent studies have shown that ROS can affect the activity of prostacycline synthase and nitric oxide synthase, then leading to NO decrease and prostaglandins. It is well reported that when mitochondria is injured and insulted, large amount of ROS can be released. However, it is still not clear whether mitochondrial damage and subsequent ROS overproduction are directly caused by contrast exposure. Study has demonstrated that, administration of CM can increase cytochrome c (Cyt c) level and significantly decrease mitochondrial membrane potential (ΔΨm) [142], which is an evidence that CM can induce severe mitochondrial injury. Significantly increased renal malondialdehyde (MDA) and catalase (CAT) levels were also observed in rats of CI−AKI, implying that contrast administration could induce oxidative stress or ROS overproduction. Taken together, studies suggest that mitochondrial injury and oxidative stress are involved in the development of CI−AKI.

Endothelin−1 (ET−1) is an important vasoconstrictor that exhibits pro-oxidant and pro-inflammatory properties [143]. ET−1 is associated with oxidative damage in endothelial cells [120]. Oxidized low-density lipoprotein can stimulate ET−1 in endothelial cells, which is responsible for ROS generation by NADPH oxidase [121]. Moreover, high ET−1 level increase the risk of CI−AKI development.

Superoxide dismutase (SOD) isoforms are the major enzymes that are expressed in kidneys, which play a critical role in inhibiting oxidative stress [144]. They can be found in both intracellular space (cytoplasm, mitochondria) and the extracellular space. The localization of SOD is different in species, but the activity of SOD is comparable in mice, humans, sheep, and other animals. As a catalysator, SOD induces the dismutation of O_2_− into O_2_ and H_2_O_2._ Therefore, SOD is regarded as the first system to fight against oxidative stress. Importantly, evidence has shown that pre-treatment with SOD can greatly prevent renal dysfunction induced by CM [145].

Vascular NADPH oxidases (NOXs) are the transmembrane enzyme complex in human vascular cells to regulate ROS generation [146]. The NOX1, NOX2, NOX4, and NOX5 are all expressed in vascular cells. Except for NOX5, NOXs have the core catalytic subunit and a few regulatory subunits [111]. Through the transfer of electrons from NADPH, molecular oxygen can generate O_2_ by NOX activation. Additionally, NOX4 has the key regulating function in generating athero-protective ROS and inhibits vascular remodeling and inflammation. Evidence showed that NOX4 is an important source of ROS responsible for CI−AKI and could be a novel potential option for prevention of CI−AKI [147].

### 4.4. Epigenetic Targets

**Figure 5:**
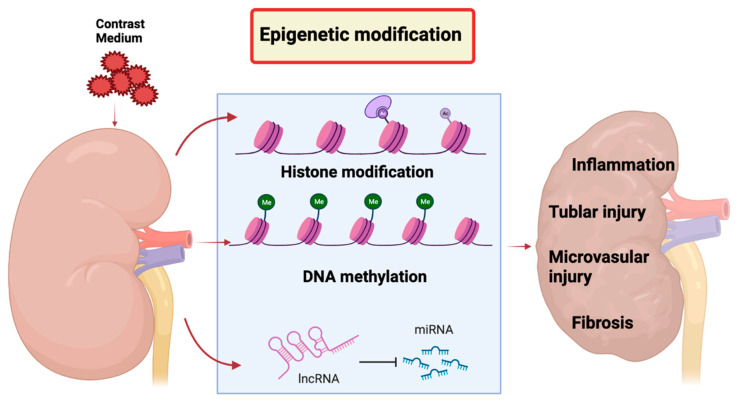
Epigenetic modifications contributing to the pathogenesis of CI−AKI. Contrast medium can induce epigenetic changes including DNA methylation, histone modification, or microRNA (miRNA) expression.

Epigenetics is characterized by any stable, heritable changes in cellular phenotype or gene expression that occurs without changes in DNA [148]. Epigenetic regulation include DNA methylation, histone modifications (methylation, acetylation, sumoylation, ubiquitinylation, phosphorylation, carbonylation, glycosylation), and microRNA (miRNA) expression [149]. Covalent modification of DNA and histones which mainly encode the epigenetic information and eukaryotic cell chromatin is important for DNA repair, replication, and transcription [150]. For instance, acetyl groups of histone proteins can be removed by histone deacetylases (HDACs) which is crucial for embryonic kidney growth and differentiation processes [149]. In pathophysiological or normal conditions, epigenetic modifications present as the vital and new contributor for the regulation of gene expression. Epigenetics regulation can occur in various human diseases and conditions, including CI−AKI (Figure 5). Numerous studies has revealed that epigenetic modification is closely related to pathogenesis of CI−AKI to regulate inflammatory response, oxidative stress, cell proliferation, and death [151,152].

DNA methylation is closely related to various diseases, including AKI. Ten eleven translocation (Tet) proteins play an essential role in various pathological processes, such as inflammation, atherosclerotic cardiovascular diseases, and leukemia via dynamic regulation of DNA methylation [153]. It has been reported that Tet2 specifically inhibits IL−6 transcription and suppresses inflammatory mediators by recruiting the histone deacetylases 2 (HDAC2) [154]. The expression of Tet2 is decreased in AKI and knockout of Tet2 promotes the production of IL−1β/Nlip3 inflammasome in AKI models [155,156]. Although the role of Tet2 in AKI is not clear, it is worth to investigate whether and how Tet2 mediated DNA methylation plays a role in CI−AKI.

Protein cores (a H3/H4 tetramer and two H2A/H2B dimers) are wrapped around by 146 base pairs that together form nucleosomes. The nucleosome is the based units of chromatin. The remodeling process has a significant role during the epigenetic modification of gene expression, the “open” and “closed” forms of chromatin. Lysine residues acetylation at the N termini of histones can eliminate positive charges and then decrease the affinity between histones and negatively charged DNA; subsequently the compact chromatin is changed to a more relaxed form for the recruitment of transcriptional activators or repressors [157]. Histone acetyltransferases (HATs) and histone deacetylase (HDACs) are responsible for catalyzing acetylation and deacetylation progress [158]. Research has reported that HATs and HDACs activation is critically associated with renal regeneration after AKI [159]. Gene for apoptosis and differentiation−1 (JASE1) isoform JADE1S and histone acetyltransferase binding to origin recognition complex-1 (HBO1) is important for epithelial cell proliferation. Acetylation of histone H4 is specifically marked by HBO1−JADE1S protein complex on lysines 5 and 12. Then, HBO1−JADE1S complex increase in the progress of epithelial cell regeneration. This study suggests that HBO1-JADE1S may play a crucial role in kidney regeneration during AKI [159], which may also be involved in the pathogenesis of CI−AKI.

Histone deacetylase 9 (HDAC9), a class IIa HDAC subtype, is overexpressed in diabetic patients with CI−AKI [160]. The upregulation of HDAC9 increases the expression of thioredoxin-interacting protein (TXNIP) and causes the activation of ASK1. It then promotes the phosphorylation of p38 MAPK substrate, eventually leading to apoptosis and oxidative stress further, promoting susceptibility of diabetic mice to CI−AKI [161]. Because the expression of HDAC9 in diabetes is more sensitive to CI−AKI, this mechanism provides a therapeutic target and diagnostic marker for the treatment of CI−AKI in diabetes.

The NAD^+^-dependent deacetylase Sirtuin 3 (Sirt3), another class 3 HDAC, is localized in the mitochondrial matrix, and is responsible for different kinds of cellular process, such as ATP generation, oxidative stress, and energy metabolism [162]. Sirt3 is overexpressed in renal tissues that can transform acetylated SOD2 (Ac-SOD2) into SOD2 to eliminate ROS. Recently, Sirt3 has been tested as an attractive target in AKI [163]. It has also been revealed that nuclear factor erythroid 2-related factor (Nrf2) plays critical role in CI−AKI [164]. Nrf2 acts as the transcription factor to regulate the transcription of antioxidant genes against various of stress response [164]. Importantly, Nrf2 plays a crucial role in the activation of the Sirt3/SOD2 signaling pathway in response to contrast-induced oxidative stress and acquired nephropathy [165].

Sumoylation and histone phosphorylation have also been associated with AKI progression [166]. Sumoylation is a post-translational modification process wherein small ubiquitin-like modifiers (SUMO) covalently bind to target proteins to regulate their function. The changes of protein sumoylation has been reported to occur dynamically in cisplatin nephrotoxic AKI mouse kidneys [167]. The inhibition of sumoylation can increase apoptosis during cisplatin contrast incubation, indicating that sumoylation has a protective effect on kidney tubular cells after injury [167]. Phosphorylation of serine 32 of H2B, 28 of H3, and serine 10 of Hx have been related to the transcription of epidermal growth factor (EGF) [168]. EGF and the EGF receptor have been reported to play significant roles in renal regeneration after AKI [169], suggesting that they may also play roles in CI−AKI.

In summary, epigenetic modifiers may be involved in the regulation of CI−AKI progression. However, the underlying mechanism of epigenetic regulation during AKI initiation and progression and the crosstalk among various epigenetic regulations in AKI, especially CI−AKI remains elusive. Further investigation about epigenetic regulations in AKI and CI−AKI is necessary, which will provide a cellular and molecular rationale for epigenetic therapy in CI−AKI.

## 5. Conclusions and Perspectives

CI−AKI is associated with the poor clinical outcomes, including increased short-term and long-term mortality, prolonged duration of hospital stays, a need for renal replacement therapy and an increase in major adverse cardiac events [169]. The etiology of CI−AKI is not fully clear. Critical predisposing factors for CI−AKI include older age, pre-existing renal failure, hemodynamic instability, congestive heart failure, diabetes mellitus, anemia and the volume of contrast media [170]. The signaling pathways involved in CI−AKI are closely associated with apoptosis, inflammation, and ROS production, and some studies suggest that these pathways may be potential targets for alleviating CI−AKI. Many more studies regarding cellular responses to contrast media are necessary.

A risk prediction tool for CI−AKI should be developed. Primarily, it might help to identify those patients at high risk for the disorder and who can then benefit by using prevention strategies. This might include intravenous isotonic saline hydration that has been proven to be effective, statins and acetylcysteine that are controversial, or other interventions targeting the risk factors that may be identified in the ongoing meta-analysis [171]. Although studies have systematically evaluated the current predictive models for CI−AKI, there has been no systematic assessment of the absolute and relative importance of the individual risk factors for CI−AKI.

In conclusion, the pathogenesis of CI−AKI and the predisposing factor for CI−AKI need to be further investigated and developed in order to provide new clinical approaches to prevent and treat CI−AKI. Although a number of experimental and clinical research has been developed for CI−AKI therapy, due to the limited understanding of the exact pathogenesis of CI−AKI, there is no significant reduction observed in CI−AKI. Given the potential of therapeutic targets in CI−AKI, the perspectives and limitations should be carefully investigated and weighed in clinical research.

## Figures and Tables

**Table 1:** Potential biomarkers of CI−AKI.

Biomarker	Position in Kidney	Function in Kidney	Reference
Semaphorin 3A	Developing glomerulus, adult podocytes and collecting tubules	Inhibitory effect on ureteric bud branching	[54]
Liver-type fatty acid-binding protein (L-FABP)	Cytoplasm of human renal proximal tubules	Regulator of fatty acid metabolism	[55]
lnc-HILPDA	Collecting tubules	Regulation of oxidative stress, vascular endothelial cell damage	[56]
lnc-PRND	Collecting tubules	Regulation of oxidative stress, vascular endothelial cell damage	[57]
Midkine (MK)	Epithelium of the proximal tubules	Regulation of oxidative stress	[58]
Cystatin C (Cys-C)	Nucleated cells, filtered by glomerulus, and reabsorbed by proximal tubule cells	Indicator of reduced kidney function	[59]
N-Acetyl-β-glucosaminidase (NAG)	Proximal tubule lysosomal enzyme	Marker of occult renal dysfunction	[60]
Kidney injury molecule-1 (KIM-1]	Upregulated in dedifferentiated proximal tubule cells	Cell phagocytosis, repair processes, and anti-inflammation, and is shed into urine	[61]
Neutrophil gelatinase-associated lipocalin (NGAL)	Expression upregulated in proximal tubule cells after renal injury	Renal tubular epithelial recovery, bacterial defense and inflammation	[62]
Interleukin-18 (IL-18)	Expressed in distal tubule cells; expression may be induced in proximal tubules	Regulator of T cell function in the kidneys during hypertension	[63]
β2-Microglobulin (β2M)	Filtered by the glomerulus and reabsorbed by the proximal tubule cells	Marker of chronic kidney dysfunction.	[64]
Retinol-binding protein (RBP)	Filtered by the glomerulus and reabsorbed by the proximal tubule cells	Marker in assessing prophylactic treatments	[65]

## Data Availability

Not applicable.
